# Effects of hypercapnia in sepsis: protocol for a systematic review of clinical and preclinical data

**DOI:** 10.1186/s13643-018-0840-4

**Published:** 2018-10-22

**Authors:** Thomas P. Clyde, Michael Coletta, Christopher Jones, Hope Kilgannon, Brian M. Fuller, Stephen Trzeciak, Brian W. Roberts

**Affiliations:** 1 The Department of Emergency Medicine, Cooper University Hospital, Cooper Medical School of Rowan University, One Cooper Plaza, K152, Camden, NJ 08103 USA; 2 The Department of Medicine, Division of Critical Care Medicine, Cooper University Hospital, Cooper Medical School of Rowan University, Camden, NJ USA; 3 Departments of Emergency Medicine and Anesthesiology, Division of Critical Care Medicine, Washington University School of Medicine, St. Louis, MO USA

**Keywords:** Hypercapnia, Hypoventilation, Mechanical ventilation, Sepsis, Septic shock, Respiratory failure, Review

## Abstract

**Background:**

Respiratory failure requiring mechanical ventilation is a common manifestation of end-organ damage among patients with sepsis and has a high morbidity and mortality rate, as well as substantial associated treatment costs. Considering the burden of this condition, there is great need to identify novel, pragmatic therapies to improve outcomes in this population. Hypercapnia has shown benefits in several different ex vivo and in vivo models of lung injury. However, it is currently unclear if hypercapnia can confer clinical benefit among patients with sepsis. The objective of this systematic review is to collate the biomedical literature of preclinical and clinical studies testing the effects of higher PaCO_2_ levels in the setting of sepsis.

**Methods:**

We will perform a qualitative systematic review of preclinical and clinical studies evaluating the effects of hypercapnia in sepsis. We will search CENTRAL, PubMed, CINAHL, and EMBASE using a comprehensive strategy*.* We will screen the reference lists of the articles we select for inclusion to identify additional studies for potential inclusion. Two independent reviewers will review all search results. Upon inclusion of articles, we will extract data using a standardized form. We will use tables to describe the study type, population included, exposure and control groups, outcome measures, and effects of exposure on outcome measures compared to controls.

**Discussion:**

This systematic review aims to synthesize the world’s literature on the effects of hypercapnia in the setting of sepsis. We expect this systematic review will find that majority of the studies will demonstrate a potential benefit of higher PaCO_2_ levels in sepsis. The results of this systematic review will contribute to the understanding of the effects of hypercapnia in the setting of sepsis and promote future research of PaCO_2_ management in mechanically ventilated patients with sepsis.

**Systematic review registration:**

The systematic review is registered in the PROSPERO international prospective register of systematic review (PROSPERO # CRD42018086703).

## Background

Sepsis places an enormous burden on healthcare systems across the globe due to its high incidence, substantial costs of treatment, and high mortality rate [1]. Patients with septic shock often develop respiratory failure, with as many as 80% requiring mechanical ventilation [2]. Sepsis patients requiring mechanical ventilation are at high risk for ventilator-associated complications [3, 4] and have a mortality rate approaching 50% [2, 5]. Considering the high incidence and mortality rate associated with mechanical ventilation among patients with sepsis, there is a need to identify novel and pragmatic approaches to improve outcomes in patients with sepsis-associated respiratory failure.

The management of the partial pressure of arterial carbon dioxide (PaCO_2_) is a fundamental aspect of care in all mechanically ventilated patients. The conventional paradigm dictates that the mechanical ventilator should be adjusted to target a normal physiological PaCO_2_ range. However, data has shown that elevated PaCO_2_ in the setting of a lung-protective ventilation strategy (i.e., permissive hypercapnia) is well tolerated and may be associated with improved survival among mechanically ventilated patients with sepsis [6]. In addition to the advantages of low stretch ventilation, preclinical data suggests hypercapnia may protect against systemic organ injury through attenuation of inflammation and free radical generation [7, 8]. The ubiquitous need to manage PaCO_2_, combined with data suggesting benefit associated with hypercapnia, suggests that PaCO_2_ could be a target to improve outcomes in this vulnerable patient population.

The aim of this systematic review is to collate the biomedical literature of (1) preclinical studies testing the effects of higher PaCO_2_ levels in the setting of sepsis and (2) clinical investigations testing the effects of hypercapnia on clinical outcomes in mechanically ventilated patients with sepsis. Our overarching hypothesis is that the majority of the studies will demonstrate a potential benefit of higher PaCO_2_ levels in sepsis. If the preponderance of data suggests hypercapnia attenuates sepsis-induced injury, then this report will provide scientific rationale for implementing high-quality interventional trials to test the effects of targeting elevated PaCO_2_ levels in mechanically ventilated patients with sepsis.

## Methods/design

### Protocol and registration

This systematic review protocol was prepared according to the guidelines set forth in the Cochrane Handbook for the Systematic Reviews of Interventions [9] and Preferred Reporting Items for Systematic Review and Meta-Analysis Protocols (PRISMA-P) 2015 (Supplemental Material) [10]. This protocol’s PROSPERO registration number is CRD42018086703.

### Search for and identification of studies

We will search CENTRAL, PubMed, CINAHL, and EMBASE using the following search terms: “hypoventilation” or “hypercapnia” or “hypercarbia” or “carbon dioxide” or “CO2” AND “bacterial infection” or “pneumonia” or “sepsis” or “septic shock” or “bacteremia.” We modeled our search terms after search terms used in previously published systematic reviews [11, 12]. We will also screen reference lists of the articles we select for inclusion to identify additional studies for potential inclusion.

### Eligibility criteria

We will include all study designs of the effects of hypercapnia in sepsis, severe sepsis, or septic shock. If a study population does not meet the inclusion criteria but a clearly defined subset of patients meets the inclusion criteria, the study may be included; however, only data pertaining to that subset of patients will be collected. The inclusion criteria for preclinical studies are as follows: (1) sepsis model (e.g., cecal ligation), (2) documented measurement of PaCO_2_, and (3) comparison of outcome measure between different PaCO_2_ levels. The inclusion criteria for clinical studies are as follows: (1) patients diagnosed with sepsis (including severe sepsis and septic shock; given we will be including paper regardless of date of publication and the variation in sepsis definitions over time, we will include any papers in which patients are diagnosed with an infection and have some definition of systemic involvement), (2) patients receiving invasive mechanical ventilation, (3) documented measurement of PaCO_2_, and (4) comparison of outcomes between different PaCO_2_ levels. We will consider studies eligible for review regardless of study design, language, or publication type. We will exclude studies that are secondary reports of studies already included. We also will exclude papers that are reviews, correspondence, or editorials; however, we will screen the reference lists of review articles to identify further studies for inclusion.

### Study selection and data abstraction

Two independent reviewers will screen the titles and abstracts of identified studies for potential eligibility. After the relevance screen, the two reviewers will compare their exclusion logs and use the kappa statistic to quantify the inter-observer agreement. In cases of disagreement, the full text will be reviewed for inclusion. All studies deemed potentially relevant will be obtained and the full manuscripts will be reviewed for inclusion. Two reviewers will independently abstract data on all patient populations, interventions, outcome measures, adverse events, and results using a standardized data collection form.

### Assessment of study bias

We will follow the Cochrane Handbook’s recommendations for assessing risk of bias in clinical and non-clinical trials [9]. For clinical trials, we will use the Cochrane Collaboration’s tool which evaluates risk of bias in six domains: selection, performance, detection, attrition, reporting, and other biases [9]. For observational human studies, we will use the New Castle-Ottawa Quality Assessment scale [9]. For animal models, we will use the Systematic Review Centre for Laboratory Animal Experimentation’s (SYRCLE) review of bias tool [13]. The overall strength of the body of evidence will be assessed using the Grading of Recommendations Assessment, Development and Evaluation (GRADE) [14].

### Analysis

We will perform a primarily qualitative analysis of the data in accordance with the recommended methodology for qualitative reviews published in the Cochrane Handbook [9]. We will first divide manuscripts into two groups: (1) preclinical and (2) clinical.

For preclinical studies, we will table the following: (1) animal model (e.g., mouse, pig); (2) study design (e.g., randomized control trial, prospective cohort); (3) mechanism for inducing sepsis (e.g., endotoxin); (4) PaCO_2_ categories; (5) outcome measures, including primary and all secondary outcomes; (6) effects of hypercapnia on outcome measures; and (7) study quality (defined above).

For clinical studies, we will table the following: (1) study design (e.g., cohort, randomized clinical trial); (2) study population (i.e., study inclusion criteria, age, and sepsis definition used); (3) PaCO_2_ categories; (4) outcome measures, including primary and all secondary outcomes; (5) effects of hypercapnia on outcome measures; and (6) study quality (defined above).

### Protocol amendments

In the event of any changes to this protocol, each amendment will be stated, a date will be provided for each amendment, and the reasoning behind each amendment will be made known.

## Discussion

Sepsis-associated respiratory failure is a common cause of morbidity and mortality, and its treatment requires an enormous amount of medical resources [1, 15]. New, cost-effective interventions are needed to address this disease process. Hypercapnia has been proposed to modulate gene expression, attenuate lung inflammation, and mitigate ventilator-associated lung injury, thereby potentially providing benefit to mechanically ventilated patients with sepsis [16–20]. To our knowledge, there is no systematic review of the world’s literature on the effects of hypercapnia in the setting of sepsis.

This systematic review aims to synthesize the world’s literature on the effects of hypercapnia in the setting of sepsis. We expect this systematic review will find that the majority of the studies will demonstrate a potential benefit of higher PaCO_2_ levels in sepsis. The results of this systematic review will contribute to the understanding of the effects of hypercapnia in the setting of sepsis and promote future research of PaCO_2_ management in mechanically ventilated patients with sepsis.

## Abbreviations

GRADE: Grading of Recommendations Assessment, Development and Evaluation
PaCO_2_: Partial pressure of arterial carbon dioxide
PRISMA-P: Preferred Reporting Items for Systematic Review and Meta-Analysis Protocols
PROSPERO: International prospective register of systematic reviews
SYRCLE: Systematic Review Centre for Laboratory Animal Experimentation
